# The Nguudu Barndimanmanha Project-Improving Social and Emotional Wellbeing in Aboriginal Youth Through Equine Assisted Learning

**DOI:** 10.3389/fpubh.2019.00278

**Published:** 2019-10-10

**Authors:** Juli Coffin

**Affiliations:** ^1^Telethon Kids Institute, Nedlands, WA, Australia; ^2^Geraldton Regional Aboriginal Medical Service, Rangeway, WA, Australia; ^3^The University of Notre Dame Australia, Broome, WA, Australia

**Keywords:** equine assisted learning (EAL), Aboriginal social and emotional wellbeing, Aboriginal youth, equine assisted therapy, Aboriginal health

## Abstract

**Background:** Recent statistics have painted a grim picture for Australia's Aboriginal youth, with reports of higher levels of almost every health indicator, including depression, sexual and emotional abuse, unemployment, and incarceration. Traditional western based therapies have proven to have limited effectiveness in engaging this group as they can often be culturally inappropriate. International studies have provided promising results using equine assisted learning, with a sound methodological basis underpinned by Indigenous ways of being and doing. In Australia Aboriginal people have strong historical ties to horses through their work on stations and were often considered some of the country's best horsemen and women. While equine assisted learning programs exist in Australia there are currently none catering specifically to Aboriginal youth, run and staffed by Aboriginal staff and provided in a culturally secure manner.

**Aims:** Alternative therapy for Aboriginal youth in the areas of grief, loss, and trauma, through an equine assisted learning program that focussed on self-concept, self-regulation, self-awareness, anxiety and depression, and sense of connectedness.

**Methods:** Participants (*N* = 270) aged 6–25 years old engaged in a minimum of 6-weeks of equine assisted learning. Each session was 45–50 min duration and occurred on a weekly basis. Sessions were undertaken individually, in pairs and in groups, depending on the needs of the participant and the focus of the session goals. Qualitative examination of the participants included photography to capture the lived experiences of the participants throughout the program. In addition an cultural and age appropriate adaptation of the Strength and Difficulties Questionnaire was trialed to track changes quantitively.

**Conclusion:** We observed improvements in self-regulation, self-awareness, and socialization skills, evident from the photography recording and the questionnaire data. In addition parent and/or caregiver and teacher reported changes in behavior, self-regulation, and socialization skills were recorded.

## Introduction

Recent statistics have painted a grim picture for Australia's Aboriginal youth, with reports of higher levels of almost every health indicator, including depression, sexual and emotional abuse, unemployment, and incarceration (1). The Western Australian Aboriginal Child Health Survey (WAACHS) reported almost a quarter of assessed Aboriginal children in WA were at a high risk of significant emotional or behavioral problems (2). When compared to rates of non-Aboriginal children (15%) this presents a considerable gap. Within WA 35.3% of Aboriginal children were reported to be living in households where a carer or carer's parent experienced forced separation from their natural family. These carer's were 1.95 times more likely to have been arrested or charged with a crime, 1.61 times more likely to report alcohol related problems in the household and 2.1 times more likely to report gambling related issues in the household (2). Not surprisingly children who were living with a carer directly affected by forced separation were at particular risk for adverse outcomes (2).

Thus, far government initiatives and various health interventions have failed to adequately address these health disparities, and often do not make allowances for the complex social, emotional, and spiritual needs of Aboriginal youth in Australia. A new approach is needed. Studies based in Canada have provided promising results in treating mental, social, emotion, and spiritual issues in Indigenous youth through the use of equine based therapy programs (3–6). These studies have provided a methodological basis that underpins Indigenous ways of being and doing, as opposed to approaching interventions aimed at improving mental health and wellbeing from a Western paradigm. In Australia equine based interventions aimed at treating mental, emotional, and social issues in at risk youth have provided promising results (7–12). However, to date there remains no equine based therapy programs aimed specifically at Aboriginal youth in Australia.

Developing new and innovative ways to engage at risk adolescents in therapy programs (or any programs for that matter) is particularly important for Aboriginal youth, due to cultural differences relating to the acceptability of traditional Westernized therapies based in clinical settings (7). Aboriginal youth may not access mental health services in Australia due to a lack of culturally secure programs, with existing services being mono-cultural and unsuitable to their needs (13). Aboriginal people have strong historical ties to horses in Australia through working and living on farms and stations, many Aboriginal people are still considered some of the country's best horsemen and women. In more remote and regional areas in particular, horses were seen as valuable assets for transport, station life, and recreation, with a good representation of Aboriginal people also involved in other facets of horsemanship both historically and in a more limited capacity currently. The Equine Assisted Learning (EAL) model provides a vehicle for Aboriginal practitioners to provide culturally secure and appropriate interventions to improve mental, emotional, social, and spiritual wellbeing (14). The physical environment in which the therapy is set allows for a more authentic experience and can help alleviate some of the mistrust and stress often felt when dealing with medical or health professionals in clinical therapeutic settings such as an office, school, in-patient facility or hospital. The physical setting of equine therapies is commonly reported as a benefit in itself, aiding in relaxation and enabling a multi-sensory approach to therapy (9).

In addition to the physical setting, recent research from Canada highlights the importance of a creating a culturally relevant space for healing to occur. Fundamentally different to Western notions of space, relating to physical distance, this space relates to collective relationships between physical, spiritual, psychological, social, economic, and cultural dimensions; all of which are foundational for healthy community (3). This inter-connectedness of all living things is essential to individual well-being and is informed by an Indigenous world view (6).

EAL involves the use of horses to engage participants in learning a variety of transferable life-skills (5), such as emotional awareness, self-regulation, healthy boundaries, improved self-awareness, and persistence (15–17). EAL utilizes a multi-disciplinary team approach usually consisting of a mental health professional (social worker, mental health nurse, counselor, or psychiatrist) and equine specialists who have addition training registration with governing bodies such as the Equine Assisted Growth and Learning Association (EAGALA), the Professional Association for Therapeutic Horsemanship (PATH International), or the Australian based Equine Psychotherapy Institute (EPI). During EAL facilitators use techniques such as metalizing (18) to highlight dysfunctional behaviors or thought-patterns that need addressing, as well as set exercises to increase trust, self-regulation, and self-awareness.

Research from an Australian based equine program that facilitates at risk youth reported high engagement of participants (12). Working with horses has also shown to be beneficial for youth who have aggressive or violent tendencies (19). Social benefits have also been reported, as equine assisted programs are frequently delivered in groups. In addition to being more cost efficient the interactive nature of EAL programs delivered in groups fosters positive social interaction, communication skills, inter-personal trust, team-work, and healthy relationship building (5, 8, 9, 20). Increased self-control and self-regulation are also commonly reported outcomes of equine assisted interventions (9, 19–21).

In addition to developing transferable life skills recent research has reported improvements in mental and emotional wellbeing in participants that have participated in EAL programs, such as increased self-esteem, self-worth (5, 21), and coping ability (22). Furthermore, decreases in anxiety (7, 23), post-traumatic stress disorder symptoms (24) cortisol levels (25), and depressive symptomology (10, 26) have been reported as a result of equine based interventions.

As Ewing (16) points out, engaging at risk adolescents is often difficult as those tasked with helping them (teachers, therapists, practitioners, and other adults) can be viewed with mistrust or suspicion. Waite and Bourke (12) acknowledge the issue of disengaged youth in treatment programs, noting that “lack of or poor engagement in therapeutic programs can mean that while clients are physically present, they do not participate, learn or benefit from a given treatment” (p. 16). EAL programs, on the other hand offer a very unique and relaxed treatment environment. One EAL participant summed up the perceived benefits of equine programs in relation to traditional therapy “… it takes away the pressure of sitting in a room, having someone watch you and ask you stupid questions” (p. 20). In addition to proving help for disengaged at-risk youth, a study on treatment for youth with substance abuse problems (a notoriously difficult to engage population) reported increased duration and completion rates for an equine based treatment therapy compared to traditional treatment (27).

The aim of the current project was to develop a pilot EAL program with at risk Aboriginal youth to determine the effectiveness of EAL in improving social and emotional wellbeing in this demographic. The primary outcome was to improve social and emotional wellbeing, participate in healthy relationships, self-awareness, and self-regulation. Secondary outcomes included increased school attendance, social and leadership skills, and positive behaviors.

## Methodology

### Community Consultation

A Community participatory action research approach was taken with this project. During discussions with the CI, who was employed as a community health researcher, and the Midwest Aboriginal Organizational Alliance (MAOA), Aboriginal youth were identified as a population of concern and an area in need of a purpose driven agenda. Education and school attendance and engagement of Aboriginal youth to stem causal pathways to drug addiction, self-harm, and mental health disorders was also identified under this youth banner. Despite identifying this priority it was concluded that no organization had previously taken the lead with a solution to addressing some of these issues through innovative intervention and implementation. The local Aboriginal Community Controlled Health Organization (ACCHO), Geraldton Regional Aboriginal Medical Service (GRAMS) therefore decided to support and invest in the training of two staff members, one of which was the CI on the project, to complete accreditation in Equine Assisted Psychotherapy Learning (EAPL). The health service also made an investment to support the program by providing top up monies and additional resources such as the use of a vehicle, public liability insurance, and staff time. The Telethon Kids Institute provided additional financial support through salary top up to enable to project to run effectively as a research pilot project to gather evidence for the use of EAL in an Aboriginal context.

The name of the program, Nguudu Barndimanmanha was decided on by local Elders, after a naming competition, which involved local youth and language experts. One of the main local languages is Wadjarri (Yamaji region) and with support from the Irra Wangga Language Center, the title nguudu barndimanmanha was decided on. This translates to “horses making good” and was reflective of the therapeutic benefits of horses. The program ran in conjunction with existing services, including GRAMS, MAOA, Bundiyarra Aboriginal Community Corporation, Geraldton Aboriginal Streetworkers Corporation, Barndimalgu Court, Midwest Psychological Association, Department for Community Corrections, Department of Child Protection (DCP), Juvenile Justice, Department of Education and the WA Police Department. Each of these services provided avenues for referral to the program, as well as individual parental referrals. In addition to the local services support, assistance was provided from Equine Assisted Psychotherapy Institute Australia particularly in the areas of evaluation and design.

### Ethics

Ethics for the project was granted through the MAOA and GRAMS Board and ethics committee. As this was only a trial it was not fully commissioned and published as a full research proposal. Any further work in this space with Aboriginal communities must respond to ethical guidelines through Western Australian Aboriginal Health Ethics Committee (or similar) and other institutional ethics boards depending on partnership involvement. This project had Elder support and was undertaken with the support of the organizations listed in the previous section under community consultation.

### Equine Program

The program ran sessions in blocks of 6–10 weeks, with each session running between 45 and 60 min. Sessions were administered in individual, pair or group formats. Every participant completed a “body check in” at the beginning of each session to promote self-awareness and self-regulation of body, emotions, thoughts and environment, regardless of format. The underpinning theoretical basis of the therapy involved Gestalt therapy techniques (28), incorporating a holistic approach to therapy which is culturally appropriate in the context of treating Aboriginal youth. The primary objective of the program was to provide an alternative (resourcing) therapy for Aboriginal youth in the areas of grief, loss and trauma. This was achieved through a focus in sessions on self-concept, self-regulation, self-awareness, anxiety and depression, and sense of connectedness. Secondary objectives included increasing school attendance through rewarding good behavior with a positive therapeutic experience, and developing a local network of mentorship, support, and education in the field of equine therapy. Elements of leadership were also incorporated as the program sought to reward positive behaviors, for example school attendance. This was integral to the sustainability of the program as it addressed many facets around the positive, particularly in the school environment, where the focus for intervention programs can often be solely based on deficits in behavior, attendance, and academic achievement. This approach minimized the potential for stigma being attached to students who attended the program.

Participants engaged in either social or emotional wellbeing (SEWB) sessions or more leadership directed sessions. The SEWB sessions were flexible in content and focus depending on the child's needs and were often focussed on building rapport, social and emotional security and safety, self-regulation, and resourcing. These sessions were always initially undertaken in a one-to-one format. Most occasions would see an individual enter the program and when ready (usually after three sessions), pair work would be introduced and finally building up to small group (3–5 participants), and occasionally whole-body awareness component (which was a mounted session, controlled on a long line in an enclosed space at the walk). Participants often got to select their desired horse/horses to work with, but the CI would choose the human aspects of partnering.

The leadership sessions were based on problem solving and looking at leadership styles and were mostly in groups of around 4–6. Horses are much attuned to leadership and seek strength but with gentleness (29). This is something that all leadership activities reflected. Mostly leadership sessions involved moving or managing the horse using different pressures and watching for responses from the horse. The participant then learnt to adjust or change their approach to elicit a different and more desired response. This skill was then transferred to a more humanistic environment of leadership. Leadership sessions were also about resourcing but had a different focus and method in the activities completed and the types of discussions held. They were suitable for small or larger group work and were good for encouraging group interactions and socialization.

### Participants

Total enrolments for the duration of the project numbered 270 participants over one calendar year (January 2015—December 2015). Participant were involved in either social and emotion wellbeing sessions (*n* = 180) or group-based work and leadership sessions (*n* = 90). Enrolment was open to youth aged 6–25 years of age, who identified as Aboriginal, and who were referred to the program through their school, the local ACCHO, parents, youth workers, or DCP. Criteria for inclusion was: Aboriginal, age based, referred, management plan in place and feedback loop established, parental/career consent and experiencing any type of behavioral or relationship issue, or involved in leadership capacity. Both males and females were involved in the program and a mix of individual and group sessions were utilized depending on progress, and initial issue/diagnosis (Table 1). Participants were not grouped into any further categories apart from school/referral pathway and age. This was due to the fact that this was a trial and there was no clear diagnosis apart from a few participants around pre-existing diagnosis of ADHD for example. Referrals would often be quite subjective and dependent upon the language and occupation specific interpretations: for example an Aboriginal Health Worker may utilize mental health language as opposed to a clinical psychologist who may use diagnostic terminology in a referral form.

**Table 1:** Participant information.

	**Males (*n* = 107)**	**Females (*n* = 163)**	**Total (*N* = 270)**
Group sessions (leadership)	20	70	90
Set sessions (6–10 week programming)	82	98	180

### Data Collection

A mixed methods approach was taken to this project, with social and emotional wellbeing changes captured by qualitative methodology while a culturally secure paper-based questionnaire was trialed to measure changes quantitatively.

Qualitative techniques utilizing photography and video were selected to capture the lived experiences of the participants as they moved through a journey of self-discovery and awareness. Qualitative methodology is focussed on gaining understanding and meaning of a person lived experience, and is highly appropriate in Aboriginal research as it seeks to understand processes and experiences, rather than measuring specific outcomes, although this can also be achieved. Photography of Aboriginal people *by Aboriginal people* provides a platform of “visual sovereignty” to explore and express Aboriginal culture, community, and experiences through visual records that are not tainted by “outsider” opinions, prejudice or perspectives (30, 31). Data collection was undertaken each session through the use of photography and videography of individual and group sessions. Time stamped still images and videos were cataloged weekly for each participants. This allowed for individual analysis of changes over time to be undertaken at the completion of the intervention. Analysis of the visual data included an examination of changes in body language and relational interactions between horse and human participants over time. In addition observations of changes in the participants' body language and self-regulation during body check-ins were noted by the CI.

In addition to the qualitative data collection CI Coffin developed and trialed a culturally secure paper based tool to assess changes in social and emotional wellbeing. The tool was based on the Strengths and Difficulties Questionnaire (SDQ) (32). The SDQ was trialed with a sample of participants however there were issues with the length, number of words and level of English literacy, and concept understanding required to successfully complete the questionnaire. The new tool was created for three distinct age groupings in a similar fashion to the SDQ (6–10, 11–16, and 17 years and over), however the questionnaire was only administered to the first two age groups due to low numbers in the third group.

A three point Likert type scale was developed (no, not sure, yes) with corresponding pictures of faces used to represent the answers for ease of use in the 6–10 year questionnaire. A five point scale was used for the 11–16 year questionnaire (no, mostly no, not sure, mostly yes, yes), with faces also used in this age group. This language developed after some trial periods into: never, not often, not sure, sometimes, always.

The final modified and simplified version of the questionnaire for 6–10 year olds included 10 question pertaining to attitudes toward school (“I feel good about coming to school”), home life (“I feel good about going home to my house and family”), social interactions (“I like to be alone at school,” “I enjoy playing at school,” “I help other students”), self-awareness and empathy (“I feel sad if someone around me is sad”) and self-regulation and confidence (“I am happy when I sit and read or draw,” “I like trying new things”).

The 11–16 year questionnaire covered aspects relating to similar categories, including learning/schooling (“I enjoy new situations and learning new things”), home life (“I enjoy going home to my family”), social interactions and self-regulation (“I try to be nice to others,” “I am helpful to others,” “I am kind to younger children”), self-awareness and psychosomatic responses (“I get a lot of tummy aches, headaches and sickness”), self-regulation (“I can sit still and apply myself to reading/art/other,” “I usually finish my work,” “I have good boundaries”), and confidence (“I do not worry about things”). Additionally several parents/caregivers (*n* = 60) filled in the SDQ parent report measure for children (PC1a) and adolescents (PY1a) (Table 2).

**Table 2:** Questionnaire data from participants.

**Test Category**	**Gender (*****n*****)**		**SDQ original**	**Pre test (before first session)**	**During (after min 5 weeks)**	**Post (end of last session)**	**Parent report/SDQ**	**Outcome**
	**M**	**F**						
6–10 years	10	10	0	20	20	20	7(PC1a)	Increase
11–17 years	5	15	6	20	18	18	5(PY1a)	Increase
>17 years	6	14	0	20	16	20		Increase
Photograph diary	60	60						
Video diary	10	20						

## Results

Attendance rates and adherence to the program were high. There were no refusals to participate or drop out of participants. Only one participant during the entire year was recorded as being absent from school on a program day and upon follow up it was revealed that his father had been released from incarceration and he was desperately wanting to see him that day. The boy attended all other sessions. School attendance was vastly improved during the program. All participants were highly engaged with the program, although there were small numbers that were initially reluctant it was never more than three sessions before positive feedback and improvements in engagement were observed, even in the most extreme cases.

### Data Collection Modality: Pre and Post Short Questionnaire

The questionnaire for the 11–16 year old groups was a 5 point Likert scale used to determine frequency of the stated behavior, ranging from never (0) to always (4). We undertook a basic analysis of the pre and post intervention results from the questionnaire data using a paired sample *t*-test to determine differences between the pre and post intervention results. Although the sample size was small (*N* = 20) we found positive results in the self-reported data for worry-related sleeplessness and incidences of anger.

**Figure 1:**
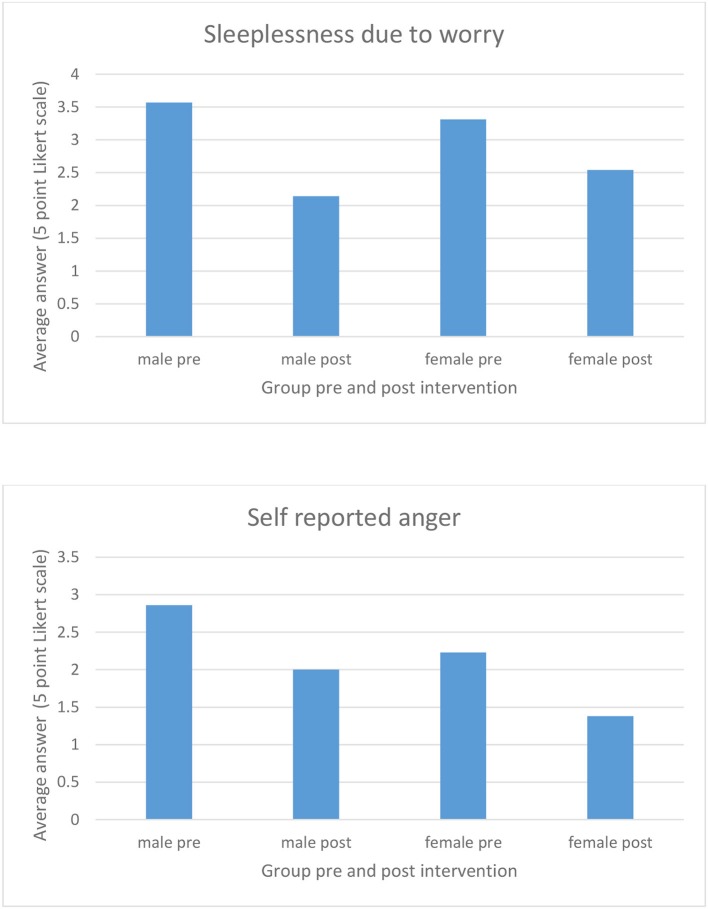
Pre and post intervention results for sleeplessness and self-reported anger domains of trial questionnaire.

For one question the participants were asked “Do you have nights where you can't sleep and lay awake worrying?” Overall the results indicated that the participants reported less frequent sleepless nights due to worrying from baseline (M = 3.4, SD = 0.99) to post intervention (M = 2.4, SD = 1.31) and this represented a significant positive change (*t* = 4.59, *p* ≤ 0.001). Examining the male (*n* = 7) participants it was found that the self-reported incidences of sleeplessness reduced from pre (M = 3.57, SD = 0.78) to post (M = 2.14, SD = 1.21) intervention (*t* = 3.87, *p* = 0.008). In the females the results also indicated a reduction in sleeplessness from pre (M = 3.30, SD = 1.10) to post intervention (M = 2.53, SD = 1.39) which was also significant (*t* = 2.99, *p* = 0.011). Males reported slightly more sleepless nights than females at baseline, and reported a larger decrease post intervention (Figure 1).

In the overall sample the incidences of feelings of self-reported anger (“I notice when I feel angry”) were lowered from pre (M = 2.45, SD = 1.19) to post intervention (M = 1.60, SD = 1.14), which was also a significant result (*t* = 7.76, *p* ≤ 0.001). When examined separately males were found to have reduced feelings of anger from pre (M = 2.85, SD = 1.06) to post (M = 2, SD = 1.15) intervention (*t* = 3.28, *p* = 0.017). Females were also found to have a reduction in frequency of anger from pre (M = 2.23, SD = 1.23) to post (M = 1.38, SD = 1.12) interventions (*t* = 8.12, *p* ≤ 0.001). Again males reported a slightly higher frequency than females at baseline however both groups reported similar changes in data from pre to post intervention.

These data represent a positive trend in self-reported behaviors, however there are several limitations to the study design. Firstly, the small numbers mean the data must be interpreted cautiously as there is limited power and secondly, the questionnaire itself was a trial to ascertain the suitability of a culturally appropriate tool. Further studies using a larger sample population are necessary to determine the suitability and reliability of the questionnaire in ascertaining behavioral changes in an Aboriginal youth population.

### Data Collection Modality: Observational, Anecdotal, and Photographic/Film

Overall observations of the photo and video collection showed vast improvements in confidence and self-regulation. This was evident in still photography analysis of posture and body language, and video recorded social interactions (see **Figures 5**, **6**). Observations of the participants included, body language feedback to the horse, self-awareness around naming feelings and behavioral aspects of feelings, increased touch seeking and contact affirmation between horses and participant, stature and body carriage, vulnerability behaviors and horses comfort level increases around mirroring participants responses. One of the other marked observable changes was the ability to increase the initial closed eyes body awareness activity and being quiet in movements/calmness around sudden and erratic responses in a physical sense.

These improvements were also noted and consolidated through feedback from teachers and parents and/or caregivers. Verbal and written feedback indicated improvements in socialization and overall focus upon returning to the classroom setting. This was particularly seen in regard to exhibiting improved anger management and self-regulation of stress and anti-social behavior. These results were echoed by parents and/or caregivers in the home environment who observed increases in positive social behaviors.

“Jonny ^*^ was never ever one to come out and sit at the table or help to do dishes, what we have noticed at a family level is his willingness to engage with us as a family now, it is a big shift for him” (caregiver of 12 years male).

In relation to family dynamics there were several boys that said repeatedly “I wish my Mum could see me now, she would be so proud, I wish she could come here.” What was interesting was the relegation of Mother to almost hero status, even when the Mother was responsible or in part for the young person's situation and circumstances.

Classroom teachers reported that many students participating were more “relaxed and happy” on their return. One teacher repeatedly stated; “I don't know what you are doing with these kids but keep doing it!” In working in conjunction with referring psychologists it was also observed from a clinical standpoint the improvements in regulation and the importance of the participant's experiences of achievement.

One student, referred from out of school on repeated home detention who suffered severe social, emotional and behavioral issues had demonstrated many outward signs of stress and trauma including severe verbal and physical ticks. This young boy (9 years old) underwent amazing transformations during sessions. Improvements in his ability to self-regulate were observed both short and long-term during the program. It was observed on collection he would be ticking, sometimes quite severely, and during the initial grounding sessions he would often struggle to even be able to close his eyes for more than 10 s. What was transformational was his complete change as soon as he was in the presence of the horses. This was captured on video. The ticking would completely disappear. He was never around horses before, and while he was never scared, he was initially wary and spent most of his sessions with the youngest mare in the herd who was not started yet under saddle and had received very limited handling apart from some halter and float exercises. From the very first contact session there was an instant connection between this boy and the horse, every time he left the mare would exhibit behaviors similar to herd separation calling out and walking the fence. Further to the changes observed during the horse sessions the young boy exhibited the highest level of self-regulation change observed during the initial grounding sessions. For 4 weeks he struggled with closing his eyes and completing the grounding exercises. By the end of the program he could close his eyes and be in the presence of the facilitator for up to 3 min.

**Figure 2:**
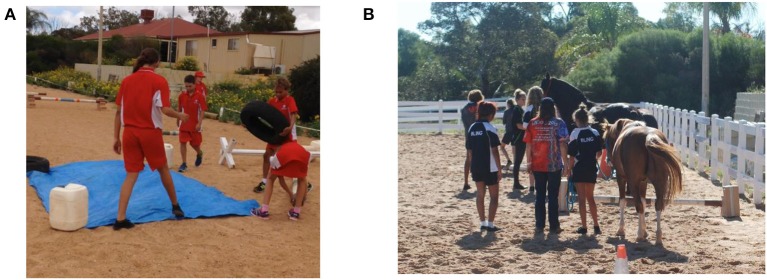
**(A,B)** Group sessions.

Improvements in socialization skills during group therapy sessions were recorded as a positive outcome as participants learnt to work together and communicate with each other to perform tasks and achieve desired outcomes (Figures 2A,B).

**Figure 3:**
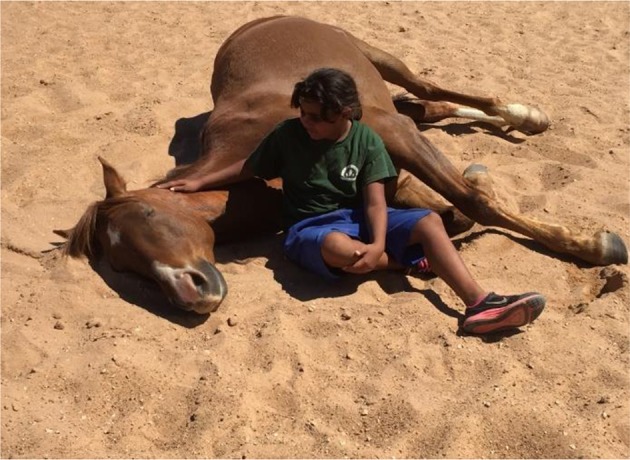
Complete trust.

In addition to the recorded changes in the participants other phenomenological occurrences were observed within the horses themselves, as they interacted and responded with participants. For example one female participant during a session mentioned she hadn't slept for days and was “just so tired.” The horse, never exhibiting such behavior prior to this interaction, laid down next to her (Figure 3), completely at ease and relaxed. The young girl went and laid on his chest and they stayed in that connection for ~20 min. This behavior had not been seen in the 7 years of owning and training that horse and has not been observed since. A horse, being a prey animal and laying down is the most vulnerable positioning that can be exhibited, this was not typical behavior unless around herd mates, in total relaxation and security.

**Figure 4:**
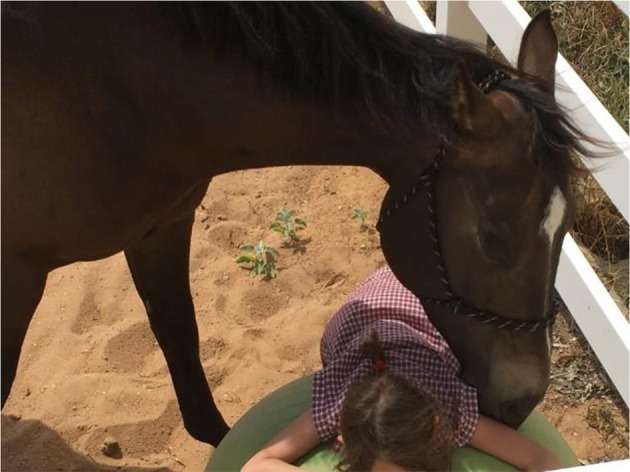
Hovering behavior from the horse.

The other phenomenological observations were also related to mirroring. In some cases participants, when asked about their current state, would say all was “well” however when they were in “real” deficit either emotionally or otherwise the horse would “hover” over them in an arc, similar to a mare or lead horse protecting and keeping an eye out for the rest of the herd or paying smaller or weaker herd members resting time (Figure 4).

**Figure 5:**
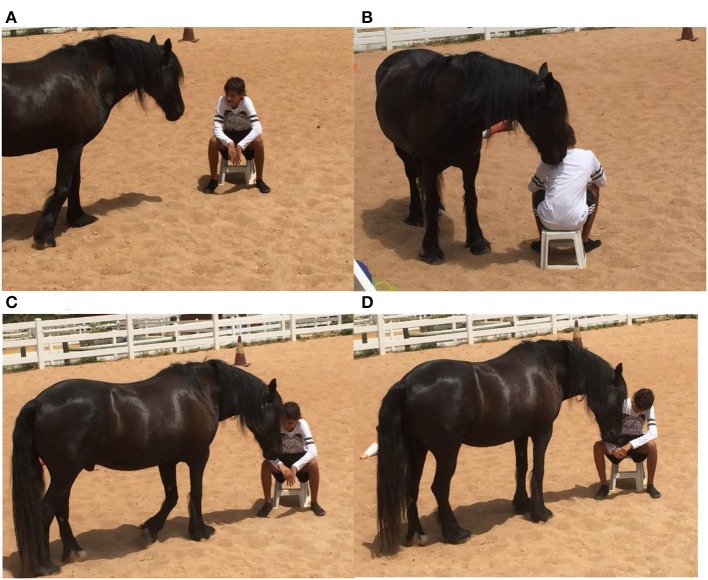
**(A)** Moving into relationship. **(B)** Initiating contact. **(C)** Reapproach from the front. **(D)** In contact and space.

**Figure 6:**
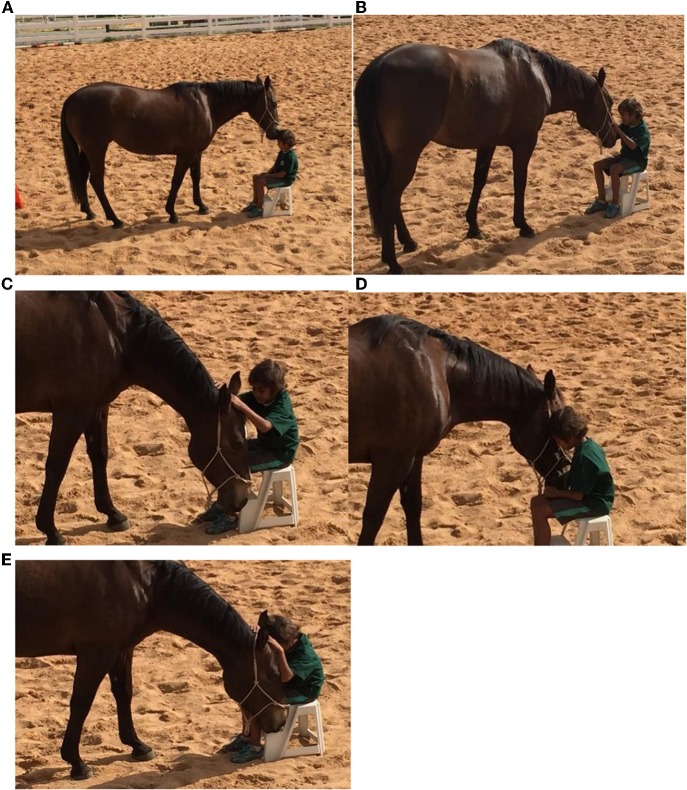
**(A)** Initial contact involving holding space, **(B)** acceptance, and **(C–E)** reciprocal relationship formation.

This mirroring phenomenon from the horses was regularly observed throughout individual sessions, as the horse moved into the participant' personal space to initiate engagement, reinforcing the desire for relationship and contact. This is an extremely affirming and positive experience as the young person encounters the horse actively seeking them out, confirming that they are a desirable and worthwhile person for the horse to be in relationship with. As can be seen in Figures 5A–D the participant is in a vulnerable position, being seated at ground level, much lower than the horse. During this activity the horse is seeking a different, more intimate relationship. When a person rides a horse there is a dominance that is inherent in both the physical act of being on top of the animal and also the act of using force of hand to move the horse where they want it to go. During EAL sessions however participants are grounded and must communicate where they want the horse to go through other methods. The larger size and power of a horse in this situation does not allow control through aggressive tactics, thus alternative forms of effective non-verbal and verbal communication (assertive rather than aggressive) need to be developed (19). In this activity the child was required to be vulnerable and allow the horse to approach them, move into their space, and accept them in a more equal relationship. This activity was used to model to the participants a safe, reciprocal relationship without dominance. Often the young people were observed in these types of interactions for prolonged moments of time (Figures 6A–E). The horse during these sessions was also observed “holding space” for the participants. This is where the horse moves close but, allows time and personal space for the child before actual touch is initiated. The horse is seeking confirmation that moving into the young person's space is ok with them before they shift closer and make contact. The participant can breathe, have their own space while still be in close proximity to the horse and gaining positive relational interaction.

Observable positive shifts toward improved SEWB occurred in all participants. Most students sent to the program due to repeated suspensions from school were able to re-engage with schooling classes and behavior markedly improved for the better after enrolling in the EAL program. Furthermore, these students were observed throughout the program and by teachers and caregivers to have elicited socialization at a higher level than previous to enrolment.

The optimum time for prevention and addressing smaller issues is of course in the younger years. The shifts seemed to be much more dramatic in comparison with adult based sessions. Each participant engaged in any of the equine assisted learning sessions always left resourced and had life skills in a real way built with a horse in facilitation but using what they already had inside themselves. The leadership sessions were very well-engaged with both from the horses and participants perspectives. Different activities around problem solving and leadership styles were very popular, and many of the girls in particular enjoyed the hands-on outdoor nature that horses provide, and the engagement levels were optimized with the use of horses where many participants across a range of sessions had never touched a horse.

## Discussion and Conclusion

This trial found conclusive evidence to support the use of EAL programs in improving the social and emotional wellbeing of Aboriginal youth. In support of previous international studies there were observed reductions in anti-social behavior (19, 33), increases in positive behaviors and body language (23, 34) and anecdotal evidence from teachers, parents, and caregivers regarding increased school attendance. Feedback from the participants also provided evidence that their experiences with the horses and the program allowed them to feel secure and safe, and they viewed the horses as accepting and non-judgemental (12). The observations around the participants desire for their parents (usually the Mother) to witness or be present during the sessions was worth noting. Findings from a community based cultural intervention for Native American youth, which utilized EAL therapy in a family setting, reported similar positive results (21). Family connections are vital and important for healthy communities and healthy individuals. This is particularly true for Aboriginal populations, who have always had strong cultural traditions around the importance of family, tradition, and culture. In Australia the historical fracturing of families has devastated many Aboriginal people and left multiple generations bearing the scars of forced separation, racially based incarceration and intergenerational trauma. Future EAL projects incorporating both parent and child could be a valuable tool in aiding the recovery of families from this trauma. Formal feedback from schools, teachers, case workers, and other key individuals would also help to strengthen the project and provide additional data for analysis of outcomes.

Drastic changes are needed to improve the unacceptable levels of self-harm, incarceration, and anti-social behavior reported in Australia's Aboriginal youth. More than that, good programmes need to be vehicles to initiate thoughts of “hope” for our young Aboriginal people. Without hope for a better future there is nothing. Changes nee to occur drastically to ensure that we do not maintain the current status of many of our young people aspiring to go to Juvenile detention or gaining hero status on their release so it becomes a normalized “rite of passage.” We must change the way young Aboriginal people are cared for and respected to be functional loving member of society. However, before we can even take that first step we need to heal the trauma that is in existence and stamp out that which is forming, whilst supporting the next generation of leaders. The Nguudu Barndimanmanha project addressed a number of important factors in respect to these issues. There are no programs that can realistically tackle all facets of an Aboriginal person's life, and it is particularly hard to engage youth, however this project has the potential to effectively address multiple concerns in large numbers of youth in a relatively short period of time. The application of EAL in this context is worthy of further investigation and future investment.

The limitations were mainly around the trial of the written questionnaire. Time restriction and the number of participants who were able to fill in the questionnaires did not allow for rigorous psychometric testing to be undertaken. Nonetheless, we were able to see an overall positive trend in the responses. In addition the participants found the modified version easier to understand and complete. A culturally secure tool for measurement of social and emotional wellbeing in an Aboriginal youth population is needed. Further testing and refining of the trialed tool with more rigorous methodology and data analysis is recommended. The addition of a control arm to allow for comparison of the EAL intervention to placebo/no intervention, or comparison of EAL to other therapies could be useful in future research. As a community based and financially supported intervention however it would not have been appropriate to withhold participation in the Nguudu Barndimanmanha program from potential participants. The decision of who was given access to a potentially beneficial intervention (and who wouldn't) may have caused undue friction in the community, who held co-ownership of the program. Therefore, a control arm was not factored into the design of this pilot project.

More time, expertise and support to develop a better baseline and recording tool would have allowed for this, but it was not the emphasis of this trial. IPads were also planned to be utilized with the questionnaires but this was also problematic with connectivity and the required funding for the number of IPads needed. Many hours of film and photo have been recorded but it would be good to validate and legitimize this as a way to collect such complex data around social and emotional well-being.

Another limitation was around the language and terminology the participants were exposed to during the program. Terms they were not generally exposed to previously, such self-regulation, emotional resourcing, and empathy were becoming familiar to the participants throughout the program. This may have allowed for “leading” when questions were asked by the facilitators pertaining to these factors.

The project had many strengths. Foremost the Nguudu Barndimanmanha project was the first of its kind in Australia, with all Aboriginal participants, and an Aboriginal led team of EAL qualified practitioners. A good mixture of male and female participants, of differing age ranges, from a number of geographical localities throughout the Midwest region allowed a broad group to be represented in the program. In addition there were students from different schools (public, private, Catholic, and Flexi-center). All referrals had written reporting about participants and follow up, allowing parents, caregivers and teachers valuable feedback and involvement in the program. Students were able to re-engage when needed once they had completed their first 6–10 week program. The use of video and photography to record changes was also beneficial as young people are very good at negotiating what they want others to hear and pretending all is okay. The camera (and horse) do not lie and therefore this was a good indicator of change, resourcing, and impact.

## Ethics Statement

This study was carried out with the recommendation of the Geraldton Regional Aboriginal Medical Service and Midwest Aboriginal Organizational Alliance board and ethics committee. The protocol was approved by the MAOA and GRAMS ethics committee. Written informed consent was obtained from all adult participants and from the parents of all non-adult participants. Additional consent to photograph participants and media release permission was obtained. All images are depicted are reproduced with informed written consent and remain the property of the study CI.

## Author Contributions

JC was the Chief Investigator on the research project and developed the methodology and protocols. She is the main contributing author of the manuscript and approved the final manuscript for submission.

### Conflict of Interest

The author declares that the research was conducted in the absence of any commercial or financial relationships that could be construed as a potential conflict of interest.
